# Online residency training during the COVID-19 pandemic: a national survey of otolaryngology head and neck surgery program directors

**DOI:** 10.1186/s40463-021-00546-6

**Published:** 2021-11-16

**Authors:** Jade Chénard-Roy, Matthieu J. Guitton, François Thuot

**Affiliations:** 1 Département d’ophtalmologie et d’oto-rhino-laryngologie – chirurgie cervico-faciale, Faculté de Médecine, Université Laval, 1050 Avenue de la Médecine, Local 4889, Quebec City, QC G1V 0A6 Canada; 2 Centre de recherche CERVO, 2601, de la Canardière, Quebec City, QC G1J 2G3 Canada; 3 Département de chirurgie, CHU de Québec-Université Laval - L’Hôtel-Dieu de Québec, 11, Côte du palais, Quebec City, QC G1R 2J6 Canada

**Keywords:** Online teaching, Otolaryngology, Residency, Resident, Program directors, Education

## Abstract

**Background:**

The COVID-19 pandemic has deeply impacted healthcare and education systems, including resident education. The impact of the pandemic on the different types of pedagogical activities, and the displacement of pedagogical activities to online modalities have not yet been quantified. We sought to evaluate the impact of the COVID-19 pandemic on formal pedagogic components of otorhinolaryngology–head and neck surgery (ORL–HNS) residency, the switch to distance learning and program director’s perceptions of the future of teaching and learning.

**Methods:**

A nationwide online survey was conducted on Canadian ORL–HNS program directors. The use of standard didactic activities in-person and online, before and during the pandemic was rated with Likert scales. Perceptions of the pandemic were described with open-ended questions.

**Results:**

A total of 11 of the 13 program directors contacted responded. The analysis were conducted using nonparametric statistics. There was a significant drop in overall didactic activities during the pandemic, regardless of the teaching format (3.5 ± 0.2 to 3.1 ± 0.3, *p* < 0.05). The most affected activities were simulation and in-house lectures. Online activities increased dramatically (0.5 ± 0.2 to 5.0 ± 0.5, *p* < 0.001), including attendance to lectures made by other programs (0.5 ± 0.3 to 4.0 ± 0.8, *p* < 0.05). Respondents stated their intention to maintain the hybrid online and in-person teaching model.

**Conclusions:**

These findings suggest that hybrid online and in-person teaching is likely to persist in the post-pandemic setting. A balanced residency curriculum requires diversity in academic activities. The pandemic can have positive consequences if higher education institutions work to better support distance teaching and learning.

**Graphical Abstract:**

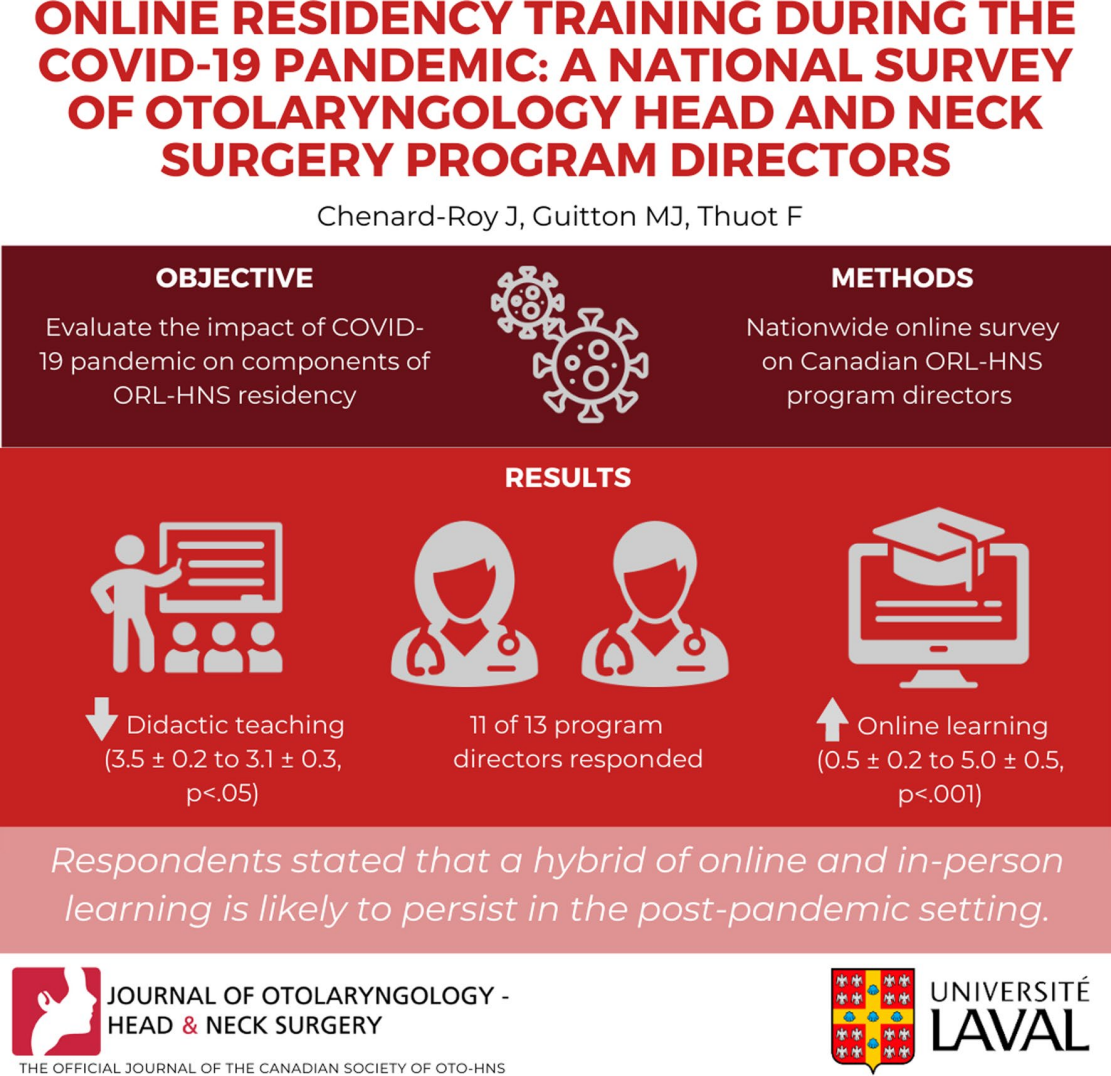

## Introduction

The first alert of a new coronavirus was declared in China, in the province of Hubei in Wuhan in December 2019, before spreading to the rest of the world. The first cases of COVID-19 were diagnosed in Canada in January 2020 and severe containment measures implemented soon after. At the time of writing this article, all formal in-person teaching activities have ceased until further notice for all Canadian medical residency programs, effective since early 2020. Although the impacts of the COVID-19 pandemic are still to be fully identified and understood, this unique sanitary crisis puts an unprecedented toll on all human activities [1–3]. While all sectors of economy have been affected by successive total or partial lockdowns all over the world, this crisis has particularly impacted healthcare and education systems. Indeed, being at the crossroads of higher education and healthcare, medical education has been particularly affected. This is also the case for residency programs. Yet, the specific manpower and expertise needs of medical training require optimal and uninterrupted training to ensure the regular arrival of new doctors in the healthcare system.

Finding the adequate balance between patient’s care, safety and education has been a prominent practical issue for residency programs since the beginning of the COVID-19 pandemic. To minimize the impacts on current and future trainees while maintaining the highest standards, academic activities in residency programs must carry on independently of local outbreaks and social distancing factors. Historically, various non-clinical teaching methods (the vast majority in-person) have been implemented by residency programs, including lectures, tumor boards, journal clubs and surgical simulation. The multiplicity of possible teaching formats gives program directors flexibility to compensate for the reduction in clinical exposure. This was manifested by an increase in didactic activities according to the perception of the resident during the first months of the pandemic [4, 5]. On the other hand, the specific impact of the pandemic on the different types of pedagogical activities has not been evaluated. Furthermore, the displacement of pedagogical activities from in-person to online has not been systematically quantified. Therefore, quantification of modality changes in these activities is necessary to identify training gaps that may arise in the new era of distance learning.

To date, a few surveys have been conducted among trainees and program directors in different medical specialties [5–7]. These surveys focused predominantly on redeployment, resident wellness and clinical exposure [7, 8]. Trainee’s clinical exposure has been heavily affected during the last months, with 98% of North American otolaryngology trainees reporting an overall decrease in clinical activities [4]. Although clinical exposure represents an important part of residency training, the exposure is nearly impossible to control in the actual endemic conditions and varies over time, geographic location and redeployment. Decrease in clinical exposure is already well described in previous surveys [7, 8]. Therefore, we intended to focus on formal pedagogic components that programs could actually control, rather than operating room and clinic exposure.

Using a nationwide survey of otorhinolaryngology–head and neck surgery (ORL–HNS) program directors of Canada, the objective of the present study was to evaluate the impact of the COVID-19 pandemic on the organization of residency programs. ORL–HNS is a specialty covering both medical and surgical aspects of its field. Given this specific nature, ORL–HNS can thus represent a good model for other specialities. We aimed at describing how residency programs changed each of their formal teaching activities to react to the pandemic crisis. Finally, we also explored the perception of program directors of the institutional availability of online and technological resources to support the pedagogical needs of their program.

## Material and methods

### Study design

We built a cross-sectional descriptive study using an online survey for ORL–HNS program directors. The main question was whether or not the use of each didactic activity for residents had changed during the pandemic. The secondary question was to assess the perception of program directors regarding the mitigation strategies that were developed.

### Questionnaire development and structure

The questionnaire used in this study was developed by a group of three researchers with expertise in an ORL–HNS residency program curriculum: two professors and one resident. Both professors were actively involved in the program, had experience in various positions on the program committee, and had teaching experience in basic sciences, clinical skills and surgery. The questionnaire was reviewed and pre-tested for format and length by an independent otolaryngologist with strong implication in an ORL–HNS residency program and with additional training in health science education. The questionnaire was originally written in French and subsequently translated in English. The English translation was then retranslated in French for control. The questionnaire was composed of three parts: (1) demographic information, (2) information related to didactic activities, and (3) open-ended questions (see “Appendix 1”).

Demographic information included the name of the university of affiliation of the program, the number of years of experience of the respondent in medical practice and as a program director and the number of residents enrolled in the program. Didactic activities were defined as formal non-clinical teaching that does not involve direct patient contact. In order to be exhaustive, we also included the various committees directly related to the operation of the program. The most common educational resources and pedagogical activities were listed in a comprehensive manner and classified in four categories: lectures, teaching activities, surgical simulation and committees (see “Appendix 1” for a complete list of identified activities). The online interface was pre-tested by an otolaryngologist blind to the study before sending out email invitations. For each activity, the frequency of face-to-face versus online use before and during the pandemic was assessed using a Likert scale (0—Never, 10—Very often). Participants were then asked to evaluate the technical support offered by their university and affiliated hospital for online distance learning tools. Provided resources, technical help for development of activities and troubleshooting support were rated using Likert scales (0—Poor, 10—Excellent). A total score for technical support was calculated based on the results to each item, with a maximum possible score of 40 points. Finally, two open-ended questions on future perspectives were included at the end of the survey in order to gather qualitative information on the anticipated changes in teaching practice post-COVID and to identify the greatest practical challenges that occurred during this period (“Appendix 1”).

### Participants

The questionnaire was sent to the 13 Canadian ORL–HNS program directors in function. Respondents were solicited electronically in English and French on July 17, 2020. The message presented information on the study scope and design as well as a link to the online questionnaire. The questionnaire was presented through LimeSurvey in both languages. The online questionnaire was preceded by a short presentation of the study. In order to ensure a maximum participation rate, up to three reminders were sent by email to the program directors between July 17, 2020, and October 20, 2020.

### Analysis

Data was extracted from LimeSurvey on Excel. Given the small size sample and to avoid issues related to normality, nonparametric tests were selected. Statistics were performed using the nonparametric Wilcoxon test. Statistical analyses were performed with SigmaPlot (version 14.5, SYSTAT Software, San Jose, CA) conducted with the rejection level set at *p* ≥ 0.05. When applicable, results are presented as means ± SEM.

For each type of pedagogic activity, the scores obtained before and during the pandemic were compared. When applicable, measures of activities that could be held both in-person and online were combined in a single mean score ± SEM (e.g., journal clubs online and in-person were combined in one mean score before and during the pandemic). Means ± SEM were also calculated for each category of activities, for all in-person or online activities.

## Results

### Participants’ characteristics

A total of 11 responses were obtained out of the 13 program directors solicited, yielding a response rate of 85%. The participants had an average of 14.3 ± 1.9 years of practice (min: 6 years, max: 28 years) and of 5.3 ± 0.7 years of experience as program director (min: 1 year, max: 9 years). Directed programs had an average of 13.3 ± 1.5 residents (min: 9 residents, max: 27 residents).

### Teaching activities

The ratings of educational resources and didactic activities by the participants before and during the pandemic are provided in Table 1. There was a statistically significant drop in overall didactic activities during the pandemic, regardless of the teaching format (3.5 ± 0.2 to 3.1 ± 0.3, *p* < 0.05). As expected, in-person activities significantly decreased (6.8 ± 0.4 to 1.1 ± 0.3, *p* < 0.001), while the online modality augmented dramatically (0.5 ± 0.2 to 5.0 ± 0.5, *p* < 0.001). While overall lectures maintained their pre-pandemic volume, in-house lectures were significantly less frequent (5.3 ± 0.2 to 3.9 ± 0.5, *p* < 0.05) in favor of an increase in attendance to online courses made by other universities (0.5 ± 0.3 to 4.0 ± 0.8, *p* < 0.05). The decrease in teaching activities also reached statistical significance (4.3 ± 0.4 to 3.6 ± 0.4, *p* < 0.05), with the journal clubs most affected even though this decline did not reach statistical significance by itself. Surgical simulation was significantly reduced during the pandemic, cadaveric dissection being the most affected modality with a substantial drop (3.1 ± 0.5 to 1.7 ± 0.6, *p* < 0.05).

**Table 1:** Mean rate of use of each didactic activity before and during the pandemic

Category	Teaching activity	Mean score (SEM)	
		Before	During
(1) Lectures	Resident presentation	**4.9 (0.1)**^**†**^	**4.5 (0.3)**^**†**^
	In-person	*9.5 (0.3)*	0.6 (0.5)*
	Online	*0.3 (0.3)*	8.3 (0.7)*
	In house lectures	**5.3 (0.2)**^**†**^	3.9 (0.5)*^,^^**†**^
	In-person	*9.8 (0.2)*	0.5 (0.5)*
	Online	*0.8 (0.3)*	7.4 (1.2)*
	Online courses made by other programs/universities	0.5 (0.3)	4.0 (0.8)*
	American online otolaryngology consortia	0.1 (0.1)	2.8 (1.0)*
	Lectures total	**3.5 (0.1)**	**3.9 (0.3)**
(2) Teaching activities	Journal club	**3.9 (0.5)**^**†**^	**2.6 (0.7)**^**†**^
	In-person	*7.7 (0.9)*	0.0 (0.0)*
	Online	*0.0 (0.0)*	5.2 (1.4)*
	Ethical issues related discussion^‡^	**3.5 (0.5)**^**†**^	**3.5 (0.5)**^**†**^
	In-person	*6.6 (0.9)*	0.9 (0.6)*
	Online	*0.3 (0.2)*	6.1 (1.0)*
	Tumor boards and other interdisciplinary clinics	**4.5 (0.4)**^**†**^	**3.7 (0.5)**^**†**^
	In-person	*8.5 (0.8)*	0.4 (0.4)*
	Online	*0.5 (0.5)*	7.0 (1.1)*
	Morbidity and mortality rounds	**5.3 (0.6)**^**†**^	**4.6 (0.7)**^**†**^
	In-person	*9.0 (0.6)*	2.4 (1.1)*
	Online	*1.5 (1.0)*	6.9 (1.0)*
	Teaching activity total	**4.3 (0.4)**	3.6 (0.4)*
(3) Surgical simulation	Cadaveric dissection laboratory	**3.1 (0.5)**^**†**^	1.7 (0.6)*^,^^**†**^
	In-person	*5.9 (0.9)*	2.8 (1.1)*
	Online	*0.3 (0.2)*	*0.5 (0.3)*
	Computer-based simulators^§^	**1.1 (0.5)**^**†**^	**1.1 (0.5)**^**†**^
	In-person	*1.0 (0.5)*	*0.7 (0.5)*
	Online	*1.3 (0.6)*	*1.5 (0.5)*
	Synthetic simulators^¶^	3.2 (0.8)	1.9 (0.7)
	Simulation total	**2.3 (0.3)**	1.5 (0.4)*

	Total in-person	6.8 (0.4)	1.1 (0.3)*
	Total online	0.5 (0.2)	5.0 (0.5)*
	**Total in-person and online**	**3.5 (0.2)**	3.1 (0.3)*

*p < 0.05 compared to pre-COVID scores

^**†**^Composite measures of activities

^**‡**^E.g.: CanMEDS, patient security, communication

^§^E.g.: Virtual reality simulators

^¶^E.g.: Mannequins, 3D printed simulators

Italics: Single measures directly questioned in the survey

Bold: Combined measures (mean) of in-person and online format, or of category of activities

### Program organization and evaluation activities

The ratings for program committees’ activity before and during the pandemic are provided in Table 2, as reported by program directors. Committees globally maintained their pre-pandemic volume. An overwhelming switch to online format was observed for both competence and residency program committees.

**Table 2:** Mean rate of activity for program committees before and during the pandemic

Committee	Mean score (SEM)	
	Before	During
Residency program committee	**4.5 (0.4)**^**†**^	**4.1 (0.6)**^**†**^
In-person	*8.8 (0.6)*	0.6 (0.6)*
Online	*0.3 (0.3)*	7.6 (1.0)*
Competence committee	**3.9 (0.6)**^**†**^	**3.9 (0.6)**^**†**^
In-person	*7.5 (1.1)*	1.3 (0.9)*
Online	*0.3 (0.3)*	6.5 (1.3)*
**Total**	**4.2 (0.4)**	**4.0 (0.6)**

*p < 0.05 compared to pre-COVID scores

^**†**^Composite measures of activities

Italics: Single measures directly questioned in the survey

Bold: Combined measures (mean) of in-person and online format, or of category of activities

### Perception of institutional technical support

The total score of appreciation for techno-pedagogical support during the pandemic varied widely between institutions, with a mean performance of 17.4 ± 3.0 (min: 4, max: 32) out of a 40 points possible score. Specifically, provided resources for distance learning were better rated for university resources (5.4 ± 3.0 out of 10) than for hospital resources (3.5 ± 3.0). Support for development of learning tools was rated poorly (4.1 ± 1.1). Technical support when facing issues with distance learning was also perceived as weak by program directors (4.5 ± 0.9).

### Qualitative analysis

Overall, participants confirmed the trend that the pandemic will durably change the teaching practices of their program (5.9 ± 0.8). 10 out of 11 commented on the anticipated changes in their teaching practices. An overwhelming proportion of respondents (8 over 10) stated that they wanted to keep a hybrid learning model (distance learning and in-person learning), or even to increase the use and accessibility of online learning opportunities after the end of the pandemic. Interestingly, the second most mentioned comment was a clear wish to increase inter-institutional teaching collaboration, notably through pan-Canadian activities (3 out of 10). Finally, only one respondent indicated that they were willing to return to the prepandemic model of 100% in-person activity education once the pandemic is over.

Out of the 11 participants, 7 commented on the greatest practical difficulties in teaching ORL–HNS during social distancing. The difficulties mentioned were highly congruent and twofold: limitations in the acquisition of technical skills, and general problems in learning process using online modalities. Difficulties in technical skill acquisition were observed or feared by 4 out 7 respondents, especially for technical skills classically acquired through laboratory practice (for instance, cadaveric dissection). The second set of difficulty was not specific to ORL–HNS but more general in the context of online teaching methods. Respondents (4 out of 7) noted difficulties of learning through online platforms, including lack of attention of the students and lack of interactions between students and teachers during online activities compared to face-to-face activities.

## Discussion

Viral transmission models predict that social distancing measures might be necessary at least until 2022, with resurgences in contagion possible in the coming years [9]. Therefore, documenting the impacts of the current crisis on residency programs and how programs adapt to the situation is relevant both for the short- and long-term future. While issues related to the well-being of residents in the context of a pandemic are of course important, it is also essential to understand the impacts of this context on the programs themselves.

For generalization purposes, otolaryngology–head and neck surgery is an interesting specialty. ORL–HNS constitutes a two-part specialty for which residents are highly trained in both the surgical and the medical fields. As a result, ORL–HNS residency programs have faced challenges relevant to both medical and surgical residency programs. According to program directors, the overall volume of pre-pandemic educational activities has not been maintained. Interestingly, this comes in contradiction with previous surveys performed on residents. Indeed, a majority of North American ORL–HNS trainees reported an increase in didactic educational activities [4]. Another study conducted on head & neck oncologic and endocrine surgery fellows reported a general increase in the use of online platforms for didactic education [5]. Several explanations can be proposed to account for this discrepancy. A significant increase has in fact been observed for interinstitutional conferences. Although residents are free to use various resources external to their program, this exposure is by definition not controlled by the program itself. Alternatively, the results of surveys on resident cohorts generally better measure residents’ perceptions rather than the reality of the program. In the context of the pandemic, the perception of the relative weight of different academic activities might have been misleading. Whatever the explanation, this clearly emphasizes the importance of consulting program leaders in addition to residents to better understand the impacts of the pandemic on educational programs.

Efforts must be made to maintain the diversity of pedagogic activities for residents. Alternatives to the in-person format exist and should be used in this regard. As stated earlier, there has been a decrease in the production of in-house lectures in favor of online lectures provided by other programs. This reduced the amount of work required from attending staff for class preparation and formal teaching. Supervisors should therefore redirect their involvement towards activities most negatively affected during a pandemic (journal clubs and simulation, for example). Data from surveys of residents revealed significant differences in their ability to participate in research activities [10]. Although this study did not explore research activities, it nevertheless demonstrates the importance of maintaining a diversity of academic activities so that they are mutually compensating.

The activities most disrupted by the shift in teaching from in-person modalities to online modalities were activities related to the acquisition of procedural skills. Procedural skills are critical for surgeons. Proficiency in these skills can be developed both in the clinical context and during simulation. Unfortunately, the feasibility of these two modalities declined dramatically during the pandemic. In a previous survey, 94% of otolaryngology residents stated that their participation in the operating room had declined [4]. Although procedural skills are acquired throughout the residency curriculum, the fine-tuning of these skills, as well as the acquisition of the advanced techniques, is usually carried out in the senior years of residency. In the vast majority of programs, the emphasis of the early years on the clinic gradually gives way to an emphasis on surgical training. In contrast, theoretical training has successfully moved to online platforms. Junior residents benefit more from a transfer to didactic activities than senior residents, as they need to acquire surgical basics and knowledge of the surgical subspecialty before they become more active in the operating room. In addition, junior residents will likely be able to at least partially recover lost surgical exposure once the pandemic is over. All residents trained during the pandemic will be affected to some extent, but the training of senior residents will likely be more affected than for junior residents. These results confirm difficulties that have been anticipated in other studies [10]. The concerns raised by program directors regarding exposure to laboratories relate to this issue of procedural skills acquisition. The importance of these concerns might be related to the fact that the ORL–HNS specialty has been targeted as one of the most at risk of contamination during the pandemic [11]. That being said, most specialities not involved in the direct management of COVID-19 patients have seen their clinical and surgical volumes be greatly impacted during the pandemic, and this challenge has likely been experienced as well. In particular, procedures speculated to be the most affected by operating room restrictions at the beginning of the pandemic were stapedectomy/ossiculoplasty, parotidectomy and congenital neck mass [10]. Further research is required to confirm these speculations and target other impacted surgical subspecialties. Program directors should thus focus on laboratory training and simulation for these surgeries and the others most affected in their own institutions.

The importance to improve inter-institutional collaborations was highlighted by the respondents. The sharing of teaching materials and lectures could indeed provide quality standardized resources to Canadian residents. Of note, such efforts have already started to be made in the USA for residents and medical students [12, 13]. In this view, several Canadian otolaryngology programs have made their Grand Rounds and other activities available to other programs.

According to program directors, online training will persist in the post-pandemic setting, combined with traditional in-person teaching. Multimodality and hybrid curricula have been outlined to be the future of training by other otolaryngology programs worldwide (USA, Germany) and also for other surgical specialities like urology [13–15].

Finally, the technical support provided by the institutions reported in this study does not seem to have followed the major shift to distance learning modalities. Significant investments are required to ensure the sustainability of programs in an era of pandemic and post-pandemic in which resident education is destined to change forever. Trainees and supervisors should be aware of the pitfalls of online learning such as computer-mediated communication exhaustion and the ways to avoid them.

Despite the relatively small size of the sample analyzed, the participation rate was high enough for the data to be representative of the ORL–HNS specialty at the national level. Therefore, the results presented here provide an accurate depiction of the actual situation in Canada, and are presumably representative of what could be seen in other Western countries. Furthermore, they might also be applied to other residency programs. Of note, this study does not explore the point of view of other academic physicians and faculty staff. Their perspectives on online teaching and changes during the pandemic would be an asset to analyze the feasibility of multimodality in the post-pandemic setting.

## Conclusion

Based on this nationwide survey of program directors, the data collected in the present study strongly suggest that a hybrid model of online and in-person teaching will persist in the post-pandemic setting. Faced with this major shift in the teaching format, attending clinicians must familiarize themselves with online educational tools. Residency programs need to recognize the challenges associated with residents’ interactivity and concentration in online activities. In order to maintain a balanced study program, the program should build on the diversity of academic activities and avoid the pitfall of focusing only on lectures. The rapid shift of residency programs to online modalities since March 2020 has been remarkable. The pandemic can have positive consequences if higher education institutions work to minimize the disadvantages of distance education while retaining its advantages. Future surveys could be conducted with program directors to reassess and describe the training changes that will effectively take place after the pandemic.

## Data Availability

The anonymized datasets used during the current study are available from the corresponding author on reasonable request.

## Appendix 1: Survey

(A) Demographic data
  - Affiliated university
  - Years as program director
  - Years of medical practice
  - Number of residents in the program
(B) Educational resources
  For each following educational resource or teaching activity: rate their use before and during the COVID-19 pandemic, using a scale from 0 to 10 (0—Never, 10—Very often).
  **Questioned educational resources**
  **Table:**
  Category		Answers	
  	Teaching activity	Before	During
  (1) Online classes	**In-house** online courses	*0–10*	*0–10*
  	Online courses made by **other programs/universities**	*0–10*	*0–10*
  	If so, from which university?	*(Open answer)*	
  	American unified online otolaryngology consortia	*0–10*	*0–10*
  (2) Teaching activity	In-person didactic lectures	*0–10*	*0–10*
  	Resident presentation		
  	In-person	*0–10*	*0–10*
  	Online	*0–10*	*0–10*
  	Journal club		
  	In-person	*0–10*	*0–10*
  	Online	*0–10*	*0–10*
  	Ethical issues related discussion (ex: CanMEDS, patient security, communication)		
  	In-person	*0–10*	*0–10*
  	Online	*0–10*	*0–10*
  	Tumor boards and other interdisciplinary clinics	*0–10*	*0–10*
  	In-person	*0–10*	*0–10*
  	Online	*0–10*	*0–10*
  	Morbidity and mortality rounds		
  	In-person	*0–10*	*0–10*
  	Online	*0–10*	*0–10*
  (3) Surgical simulation	Cadaveric dissection laboratory		
  	In-person	*0–10*	*0–10*
  	Online	*0–10*	*0–10*
  	Computer-based simulators (ex: Virtual reality simulators)		
  	In-person	*0–10*	*0–10*
  	Online	*0–10*	*0–10*
  	Synthetic simulators (ex: Mannequins, 3D printed simulators)	*0–10*	*0–10*
  (4) Committees	Residency program committee	*0–10*	*0–10*
  	Competence committee	*0–10*	*0–10*
  (III) Technical support
    Rate the performance of your faculty/hospital for each of the following resources on a scale from 0 to 10 (0—Poor, 10—Excellent).
    - **University** provided resources for distance learning during the pandemic.
    - **Hospital** provided resources for distance learning during the pandemic.
    - Hospital/university provided **technopedagogical support** to develop distance learning tools during the pandemic.
    - Hospital/university provided **technical support** when facing technical issues with distance learning during the pandemic.
  (IV) Open-ended questions
    - To what extent are you **worried** about **formal learning** in your program if the social distancing measures persist? On a scale from 0 to 10 (0—Not at all, 10—Very worried)
    - If the pandemic was to end completely today, to what extent would you **change your teaching practices** compared to before the pandemic? On a scale from 0 to 10 (0—Not at all, 10—Highly likely).
    - If you were to change your practices, how would you do it? (Open answer)
    - In your opinion, what are the **greatest practical difficulties** in teaching otolaryngology during social distancing? (Open answer)
